# Supplementary material for: Systems Biology of Cold Adaptation in the Polyextremophilic Red Alga Galdieria sulphuraria
Source: Front Microbiol. 2019 May 1;10:927. doi: 10.3389/fmicb.2019.00927 (PMC6504705; doi:10.3389/fmicb.2019.00927)
Supplement: Supplementary file 1 [file Data_Sheet_1.PDF]

SUPPLEMENTARY MATERIAL

Supplementary Table S1: DNA-Seq and mapping statistics. 150x2 bp paired-end reads were generated through Illumina ® HiSeq3000 sequencing and mapped to the genome of *G. sulphuraria* RT22 using bwa. **Sample ID**: Unique ID of the sequenced sample. **Tot. Reads**: Numer of generated reads. **Aligned Reads**: Number of aligned reads. **% Aligned Reads**: Percentage of aligned reads. **Indel rate**: Ratio of InDels per aligned read. **Mismatch rate**: mismatches per read. **Paired Alignment rate**: Read pairs concordantly aligned on the same contig, taking into account their insert size. **Genome Coverage**: average genome per base coverage.

Sample ID	Tot. Reads	Aligned Reads	% Aligned Reads	Indel rate	Mismatch rate	Paired Alignment rate	Genome Coverage
42°C_9	40748234	40092215	98.39%	0.000276	0.0056	0.997197	384.85
42°C_6	57701476	56813602	98.46%	0.000283	0.0058	0.996572	545.36
42°C_3	47770978	47091507	98.58%	0.000229	0.0052	0.997448	452.03
42°C_1	43302404	42727881	98.67%	0.00026	0.0056	0.997356	410.15
28°C_6	46621914	45959186	98.58%	0.000257	0.0054	0.997272	441.16
28°C_5	47371438	46478585	98.12%	0.000267	0.0055	0.997041	446.15
28°C_4	49079250	48317389	98.45%	0.000273	0.0057	0.99693	463.80
28°C_3	45309302	44473029	98.15%	0.00027	0.0057	0.996947	426.90
28°C_2	31506428	30594494	97.11%	0.000264	0.0056	0.997038	293.68
28°C_1	61268880	60321126	98.45%	0.000303	0.0062	0.996813	579.02

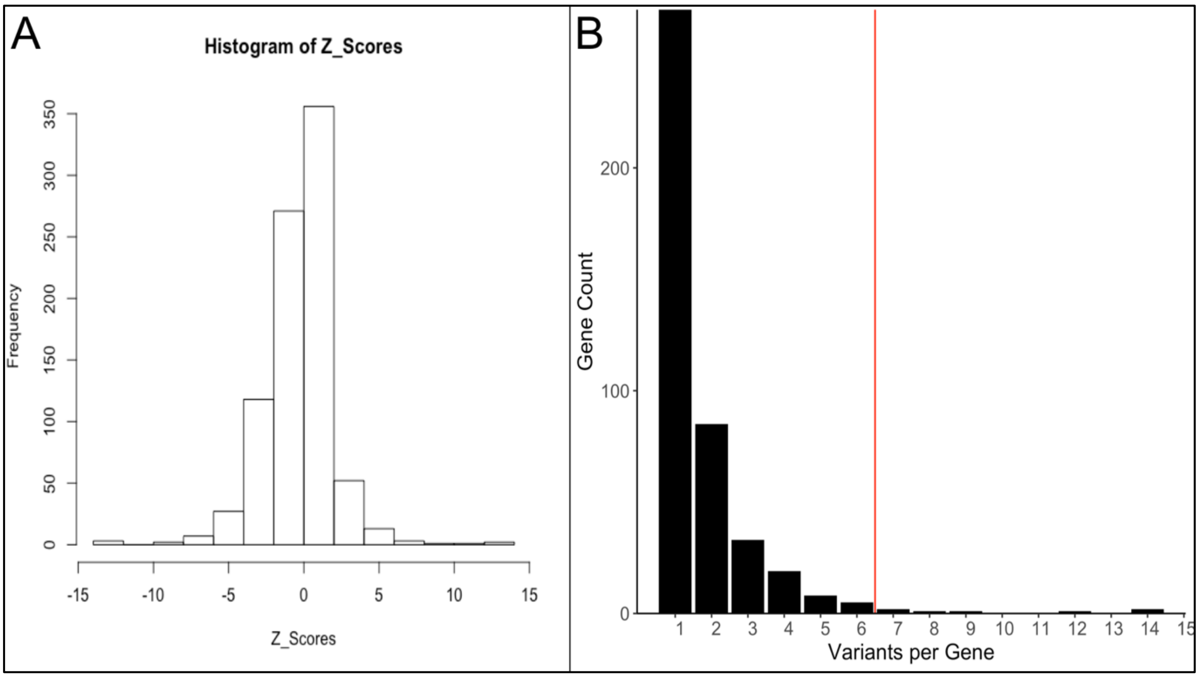

Supplementary Figure S1 – Z-score distribution of variants per gene. **A:** The calculation of Z-Scores requires a normal distribution of the data in order to calculate standard deviation. To calculate standard deviation, we mirrored the positive distribution of variants per gene by mirroring the number of variants per gene for each gene at x = 0. Doing so, we obtained a normal distribution (Shapiro-Wilk normality test, $p > 0.05$). **B**: Genes on the right side of the red line belong to the 99th percentile of genes with the most variants per gene. Z-scores above 2.56 translated into 7 or more variants per gene.

Supplementary Listing S1A - Variant hotspots

Gsulp_RT22_67_G1995: (14 variants, Z-score = 5.94) is a tyramine oxidase using copper and 2,4,5-trihydroxyphenylalanine (TPQ) as cofactors which is involved in the metabolism of tyramine, a biogenic amine acting as signal in eukaryotes where it fulfills signaling functions including cell differentiation and growth and detoxification (Kumar et al., 1996) (Signalling, Cell Cycle). Its activation/deactivation is highly regulated through various processes that can target the quinone cofactor or specific amino acid residue of the target enzyme (Longu et al., 2005).

Gsulp_RT22_116_G6539: (14 variants, Z-score = 5.94) is a hypothetical protein which is possibly involved in protein binding (GO:0005515, only available annotation).

Gsulp_RT22_67_G2013: (12 variants, Z-score = 5.10) is an APC family amino acid permease involved in the transmembrane transport of amino acids into the cell (Transport) that was potentially acquired via HGT. While temperature affects the maximum uptake rates of amino acids, it does not express a major effect on protein inhibition (Horák and Kotyk, 1977). Of the 12 variants affecting this gene, only two are synonymous. We observe a trend from bigger to smaller AA (p.Thr394Ser, p.Ile343Val, p.Ile279Val, p.Ser365Gly, p.Ile257Val, p.Thr349Ala) and changes in polarity/charge (p.Asn123Lys, p.Asn123Asp, p.Glu122Gln).

Gsulp_RT22_107_G5273: (9 variants, Z-score = 3.82) is an armadillo/beta-catenin repeat family. Armadillo repeats are contained in proteins such as catenin, importin and plakoglobin, and others (Peifer et al., 1994; Huber et al., 1997). β-catenin, the best-characterized protein containing armadillo repeats, is a dual function protein acting as a subunit of the cadherin protein complex (cell-cell adhesion, binding actin filaments across cells) and as an intracellular signal transducer of Wnt signaling (growth control) during embryonic development (Nusse and Clevers, 2017).

Gsulp_RT22_99_G4499: (8 variants, Z-score = 3.40) is the molecular chaperone DnaJ, also known as Hsp40. Various Hsp40's act as cochaperones of Hsp70 and regulate its localizations as well as affinity towards complexation with client proteins (Fan et al., 2003).

Gsulp_RT22_112_G5896: (7 variants, Z-score = 2.97) encodes the mitochondrial or chloroplast 50S ribosomal protein L1 involved in the translocation of deacylated tRNAs from the P to the E site (Valle et al., 2003). Both the correct biogenesis of ribosomes as well as the process of ribosomal translation are highly temperature dependent and necessitate proper

chaperone function, such as DnaJ/Hsp40 (Al Refaii and Alix, 2009), which we also found to be a mutation hotspot in this dataset.

Gsulp_RT22_116_G6590: (7 variants, Z-score = 2.97) is a LYR family protein. The Nfs1 homolog in yeast encodes for an interacting protein essential in the early steps of Fe/S cluster biogenesis in mitochondria by interacting with Nfs1, the mitochondrial cysteine desulfurase, thus modulating Fe/S cluster assembly (Adam et al., 2006).

Supplementary Listing S1B - Further non-random genic variants

Gsulp_RT22_82_G3036: (1 "Cold" variant) has obtained a single intron variant (c.1761+13C>T) which is present in all samples collected at 28°C (28°C_1 – 28°C_6), but absent from all samples taken at 42°C (42°C_1 – 42°C_9). Hence it was obtained within the first 16 generations of growth at cold temperatures. The annotation of this gene suggests the GTPase-activating ADP-ribosylation factor ArfGAP2/3 (Glo3 in yeast, AGD8/9/10 in *A. thaliana*) (Estrada et al., 2015). ArfGAP2/3 is paramount for regulating the activity amplitude of Arf1 (signaling). Arf1 is a small GTPase central for the generation of vesicles and the correct transport localization of various compounds from the trans-Golgi network to the plasma membrane (Min et al., 2013). In temperature context, Δglo3 mutants in yeast displayed growth defects at 15°C (Poon et al., 1999). Unless affecting splice site and other structural components, intronic mutations may affect gene expression levels. Hence, this mutation may add a beneficial transcriptional activity on ArfGAP2/3 which in turn controls the amplitude of Arf1.

Gsulp_RT22_83_G3136: (2 "Cold" variant, 4 variants in total) accumulated two intron variants (c.416-19_416-18insG, c.416-22_416-21insT), both measured at the fifth sampling timepoint at 28°C (28°C_5, 67 – 85 generations). Changes in transcription levels of the gene may be assumed. The gene encodes a nuclear polyadenylated RNA-binding NAB3/HDMI protein functioning as transcription termination factor modulating the transcriptional termination at the 3' end of target genes, thus affecting their transcriptional regulation (the underlying mechanism is not fully understood). In yeast, NAB3/HDM1 is also connected to the longevity of CLN3 mRNA (Sugimoto et al., 1995). CLN3 is a G1 cyclin that functions as a rate-limiting activator of cell cycle initiation (gene regulation, signaling) which affects the transcription of NAB3/HDM1 thus indirectly influences the temperature dependent cell cycle regulation.

Gsulp_RT22_64_G1844: (2 “Cold” variants, 4 variants in total) is a peptidylprolyl cis-trans isomerase (cyclophilin), possibly located in the chloroplast, containing a U box domain (Aravind and Koonin, 2000). The U box domain indicates that the protein can be posttranslationally modified through targeted ubiquitination. Cyclophilins occur in all subcellular compartments and are involved in processes such as protein maturation by catalyzing protein folding, receptor complex stabilization, receptor signaling, apoptosis, RNA processing and spliceosome assembly (gene regulation, signaling) (Romano et al., 2004). Both variants were obtained in the last 28°C timepoint (28°C_6, 86 – 101 generations) and are transversions located in the 5’UTR sequence, one of which (c.-18A>T) leads to the gain of a premature start codon increasing the protein length by 6 amino acids.

Gsulp_RT22_118_G6841: (1 “Cold” variant) was annotated as arabinose-proton symporter belonging to the MFS family of sugar transporters, which are not directly affected by temperature (temperature only affects V_{max}, not K_m) (Law et al., 2008). This gene gained one missense variant (p.Trp209Leu) at the fourth 28°C sampling time point (28°C_4, 50 – 66 generations). While tryptophan is bulkier than leucine, both amino acids are hydrophobic.

Gsulp_RT22_65_G1905: (1 “Cold” variant, 3 variants in total) was annotated as inositol-2-dehydrogenase (myo-inositol) catalyzing the oxidation of the carbocyclic myo-inositol [myo-inositol + NAD^+ ⇌ 2,4,6/3,5-pentahydroxycyclohexanone + NADH + H^+] (Larner et al., 1956). It is probably located on clathrin vesicles. Clathrin vesicles are generated by the complete invagination of clathrin-coated pits on the plasma membrane which leads to the formation of cytosolic vesicles. Hence, they play a relevant part in endocytosis, by which extracellular material can be trapped into vesicles and imported into the cell (Royle, 2006). The temperature relevant mutation is a missense variant (p.Asn277Ser) occurred at the fourth 28°C sampling time point (28°C_4, 50 – 66 generations). Both Asn and Ser are small polar amino acids with uncharged side chains.

Gsulp_RT22_79_G2795: (1 “Cold” variant) was annotated as Iota DNA polymerase/DNA polymerase IV which gained a missense variant (p.Pro443Ser) in the last sampling point of 28°C (28°C_6, 86 – 101 generations). It is an error-prone DNA directed DNA polymerase which is activated upon SOS signaling as promoting adaptive point mutations as part of the coordinated cellular response to environmental stress (Napolitano et al., 2000; McKenzie et al., 2001). While proline is a unpolar amino acid with an aliphatic side chain, serine is polar.

Gsulp_RT22_118_G6798: (1 "Hot" variant, 3 variants in total) was annotated as P-loop/NTPase domain-containing 2-phosphoglycerate kinase. 2-phosphoglycerate kinase catalyzes the first synthesis step of the unusual compound cyclic 2,3-diphosphoglycerate (cDPG) (Lehmacher and Hensel, 1994). cDPG is a compatible solute that has been found increasing the optimal growth temperature of hyperthermophile methanogens as well as salt stress halotolerant microorganism (Santos and da Costa, 2002; Roberts, 2005). The variant was measured at the sixth 42°C sampling time point (42°C_6, 54 – 109 generations) and constitutes a missense variant (p.Met747Val).

Gsulp_RT22_71_G2297: (1 "Hot" variant) is a hypothetical protein located in membranes that gained an intron variant (c.856+11T>A), possibly influencing its expression pattern, at the third 42°C sampling time point (42°C_3, 16 – 54 generations).

Gsulp_RT22_84_G3173: (1 "Hot" variant) is a hypothetical protein located in membranes that gained a missense variant (Arg115Gln) at the sixth 42°C sampling time point (42°C_6, 54 – 109 generations).

Gsulp_RT22_85_G3288: **(**1 "Hot" variant**)** gained an in-frame insertion variant (p.Arg302dup) at the ninth 42°C sampling time point (42°C_9, 109 – 162 generations). It was annotated as chloroplastic/mitochondrial Phosphoribosylformylglycinamidine synthase (FGAM synthase) which catalyzes the fourth step of purine biosynthesis (Zhang et al., 2008).

Gsulp_RT22_67_G1991: **(**1 "Hot" variant**)** was annotated as a xanthine/uracil/vitamin C permease transmembrane transporter that is potentially subjected do intrinsic misfolding (Krypotou et al., 2015). The majority of members belonging to this family are uncharacterized and potentially transport other substrates. It gained a missense variant (p.Ile584Met) during the ninth 42°C sampling time point (42°C_9, 109 – 162 generations). As temperature does not directly affect transport protein functionality, we suspect an adaptation towards altered membrane viscosity.

Gsulp_RT22_67_G2027: (1 "Hot" variant, 4 variants in total) was annotates as the interconvertible, molybdenum cofactor-dependent enzyme xanthine dehydrogenase/oxidase (XDH/XO) which catalyzes the oxidation of hypoxanthine (natural purine derivative) to xanthine and the subsequent oxidation of xanthine to uric acid while generating superoxide

and hydrogen peroxides as by-products (Leimkühler and Klipp, 1999; Nishino et al., 2008). The variant (missense, p.Val797Ala) was measured in the ninth 42°C sampling time point (42°C_9, 109 – 162 generations).

Gsulp_RT22_99_G4476: (2 "Hot" variants, 3 variants in total) was annotated as γ-tubulin ring complex 3 protein component homologous to Spc98. Spc98 is connected to the recruitment of tubulin at the nuclear surface and possibly along multiple microtubule-nucleating sites in plants (Gunawardane et al., 2000; Erhardt et al., 2002). Both variants are frameshift variants that affect the same position (c.2134dupT, c.2133_2134insA). Both lead to a premature stop codon after changing the terminal amino acids to FLERVVK, leading to a loss of trailing 143 amino acids out of its total 2580.

Gsulp_RT22_107_G5261: (1 "Cold" variant, 4 variants in total), Gsulp_RT22_79_G2814 (1 "Cold" variant), Gsulp_RT22_104_G4978 (1 "Hot" variant) and Gsulp_RT22_65_G1893 (1 "Hot" variant, 6 variants in total) gained synonymous variants each and were not further considered.

Adam, A.C., Bornhovd, C., Prokisch, H., Neupert, W., and Hell, K. (2006). The Nfs1 interacting protein Isd11 has an essential role in Fe/S cluster biogenesis in mitochondria. *EMBO J* 25(1)**,** 174-183. doi: 10.1038/sj.emboj.7600905.

Al Refaii, A., and Alix, J.H. (2009). Ribosome biogenesis is temperature-dependent and delayed in Escherichia coli lacking the chaperones DnaK or DnaJ. *Molecular microbiology* 71(3)**,** 748-762.

Aravind, L., and Koonin, E.V. (2000). The U box is a modified RING finger — a common domain in ubiquitination. *Current Biology* 10(4)**,** R132-R134. doi: 10.1016/s0960-9822(00)00398-5.

Erhardt, M., Stoppin-Mellet, V., Campagne, S., Canaday, J., Mutterer, J., Fabian, T., et al. (2002). The plant Spc98p homologue colocalizes with γ-tubulin at microtubule nucleation sites and is required for microtubule nucleation. 115(11)**,** 2423-2431.

Estrada, A.F., Muruganandam, G., Prescianotto-Baschong, C., and Spang, A. (2015). The ArfGAP2/3 Glo3 and ergosterol collaborate in transport of a subset of cargoes. *Biol Open* 4(7)**,** 792-802. doi: 10.1242/bio.011528.

Fan, C.Y., Lee, S., and Cyr, D.M. (2003). Mechanisms for regulation of Hsp70 function by Hsp40. *Cell Stress Chaperones* 8(4)**,** 309-316.

Gunawardane, R.N., Martin, O.C., Cao, K., Zhang, L., Dej, K., Iwamatsu, A., et al. (2000). Characterization and reconstitution of Drosophila gamma-tubulin ring complex subunits. *J Cell Biol* 151(7)**,** 1513-1524.

Horák, J., and Kotyk, A. (1977). Temperature effects in amino acid transport by Saccharomyces cerevisiae. *Experimental Mycology* 1(1)**,** 63-68.

Huber, A.H., Nelson, W.J., and Weis, W.I. (1997). Three-dimensional structure of the armadillo repeat region of beta-catenin. *Cell* 90(5)**,** 871-882.

Krypotou, E., Scazzocchio, C., and Diallinas, G. (2015). Functional characterization of NAT/NCS2 proteins of Aspergillus brasiliensis reveals a genuine xanthine-uric acid transporter and an intrinsically misfolded polypeptide. *Fungal Genet Biol* 75**,** 56-63. doi: 10.1016/j.fgb.2015.01.009.

Kumar, V., Dooley, D.M., Freeman, H.C., Guss, J.M., Harvey, I., McGuirl, M.A., et al. (1996). Crystal structure of a eukaryotic (pea seedling) copper-containing amine oxidase at 2.2 å resolution. *Structure* 4(8)**,** 943-955. doi: 10.1016/s0969-2126(96)00101-3.

Larner, J., Jackson, W.T., Graves, D.J., and Stamer, J.R. (1956). Inositol dehydrogenase from Aerobacter aerogenes. *Arch Biochem Biophys* 60(2)**,** 352-363.

Law, C.J., Maloney, P.C., and Wang, D.N. (2008). Ins and outs of major facilitator superfamily antiporters. *Annu Rev Microbiol* 62**,** 289-305. doi: 10.1146/annurev.micro.61.080706.093329.

Lehmacher, A., and Hensel, R. (1994). Cloning, sequencing and expression of the gene encoding 2-phosphoglycerate kinase from Methanothermus fervidus. *Mol Gen Genet* 242(2)**,** 163-168.

Leimkühler, S., and Klipp, W. (1999). Role of XDHC in molybdenum cofactor insertion into xanthine dehydrogenase of Rhodobacter capsulatus. *Journal of bacteriology* 181(9)**,** 2745-2751.

Longu, S., Mura, A., Padiglia, A., Medda, R., and Floris, G. (2005). Mechanism-based inactivators of plant copper/quinone containing amine oxidases. *Phytochemistry* 66(15)**,** 1751-1758. doi: 10.1016/j.phytochem.2005.06.013.

McKenzie, G.J., Lee, P.L., Lombardo, M.-J., Hastings, P., and Rosenberg, S.M. (2001). SOS mutator DNA polymerase IV functions in adaptive mutation and not adaptive amplification. *Molecular cell* 7(3)**,** 571-579.

Min, M.K., Jang, M., Lee, M., Lee, J., Song, K., Lee, Y., et al. (2013). Recruitment of Arf1-GDP to Golgi by Glo3p-type ArfGAPs is crucial for golgi maintenance and plant growth. *Plant Physiol* 161(2)**,** 676-691. doi: 10.1104/pp.112.209148.

Napolitano, R., Janel-Bintz, R., Wagner, J., and Fuchs, R. (2000). All three SOS-inducible DNA polymerases (Pol II, Pol IV and Pol V) are involved in induced mutagenesis. *The EMBO journal* 19(22)**,** 6259-6265.

Nishino, T., Okamoto, K., Eger, B.T., Pai, E.F., and Nishino, T. (2008). Mammalian xanthine oxidoreductase - mechanism of transition from xanthine dehydrogenase to xanthine oxidase. *FEBS J* 275(13)**,** 3278-3289. doi: 10.1111/j.1742-4658.2008.06489.x.

Nusse, R., and Clevers, H. (2017). Wnt/beta-Catenin Signaling, Disease, and Emerging Therapeutic Modalities. *Cell* 169(6)**,** 985-999. doi: 10.1016/j.cell.2017.05.016.

Peifer, M., Berg, S., and Reynolds, A.B. (1994). A repeating amino acid motif shared by proteins with diverse cellular roles. *Cell* 76(5)**,** 789-791.

Poon, P.P., Cassel, D., Spang, A., Rotman, M., Pick, E., Singer, R.A., et al. (1999). Retrograde transport from the yeast Golgi is mediated by two ARF GAP proteins with overlapping function. *EMBO J* 18(3)**,** 555-564. doi: 10.1093/emboj/18.3.555.

Roberts, M.F. (2005). Organic compatible solutes of halotolerant and halophilic microorganisms. *Saline Systems* 1(1)**,** 5. doi: 10.1186/1746-1448-1-5.

Romano, P.G., Horton, P., and Gray, J.E. (2004). The Arabidopsis cyclophilin gene family. *Plant Physiol* 134(4)**,** 1268-1282. doi: 10.1104/pp.103.022160.

Royle, S.J. (2006). The cellular functions of clathrin. *Cell Mol Life Sci* 63(16)**,** 1823-1832. doi: 10.1007/s00018-005-5587-0.

Santos, H., and da Costa, M.S. (2002). Compatible solutes of organisms that live in hot saline environments. *Environmental Microbiology* 4(9)**,** 501-509. doi: 10.1046/j.1462-2920.2002.00335.x.

Sugimoto, K., Matsumoto, K., Kornberg, R.D., Reed, S.I., and Wittenberg, C. (1995). Dosage suppressors of the dominant G1 cyclin mutantCLN3-2: Identification of a yeast gene encoding a putative RNA/ssDNA binding protein. *Molecular General Genetics* 248(6)**,** 712-718.

Valle, M., Zavialov, A., Sengupta, J., Rawat, U., Ehrenberg, M., and Frank, J. (2003). Locking and unlocking of ribosomal motions. *Cell* 114(1)**,** 123-134. doi: Doi 10.1016/S0092-8674(03)00476-8.

Zhang, Y., Morar, M., and Ealick, S.E. (2008). Structural biology of the purine biosynthetic pathway. *Cell Mol Life Sci* 65(23)**,** 3699-3724. doi: 10.1007/s00018-008-8295-8.
